# Clinical and Microbiological Profiles of Shigellosis in Children

**Published:** 2014-12

**Authors:** A.V. Sangeetha, Subhash Chandra Parija, Jharna Mandal, Sriram Krishnamurthy

**Affiliations:** ^1^Department of Microbiology, JIPMER, Puducherry, India; ^2^Department of Paediatrics, JIPMER, Puducherry, India

**Keywords:** Children, CTX-M, ESBL, Shigellosis, TEM, Virulence genes

## Abstract

Shigellosis presents with varied clinical features are dictated by the species involved, virulence factors of the strain, and the host immune status. We studied the species, virulence genes, and antibiotic susceptibility pattern of the *Shigella* strains isolated from 33 children aged less than 12 years, with clinical features of shigellosis. Identification and antibiotic sensitivity of *Shigella* species were done using disc diffusion and E-test. Multiplex PCR was done for the detection of virulence genes (*ipa*H, *ial*, *set*1A, *set*1B, *sen*, and *stx*) and ESBL genes. Parents of the children were interviewed using structured questionnaire to assess the severity of the disease; 26 (79%) of the isolates were *Shigella flexneri.* Ciprofloxacin and ceftriaxone resistance was seen in 23 (69%) and 3 (9%) *Shigella* isolates respectively. Two ceftriaxone-resistant strains were found to harbour *bla*CTX gene and the third *bla*TEM gene. Virulence gene *ipa*H was detected in 100% of strains while *ial*, *sen*, *set*1A, and *set*1B were detected in 85%, 61%, 48%, and 48% respectively.

## INTRODUCTION

Diarrhoeal disease continues to be a leading cause of morbidity and mortality worldwide. Despite the advances occurring in water treatment and sanitary conditions, diarrhoea continues to remain a leading public-health problem worldwide. Also, there is an increased recognition in the importance of *Shigella* species as an enteric pathogen due to its potential devastating consequences resulting from strains resistant to all available antimicrobial agents. Shigellosis, in the past, was quite different from the current scenario. *Shigella dysenteriae,* once the most common species causing severe disease, has been replaced by *Shigella flexneri* in most of the countries (1).

The burden of shigellosis in a tertiary-care hospital of north India over a period of six years was found to be around 4% (2). The clinical spectrum of shigellosis in children ranges from mild, self-limiting, non-inflammatory diarrhoea to severe, inflammatory, bloody diarrhoea with fever, abdominal cramps, and extra-intestinal complications. Several mechanisms have been proposed to explain the varied clinical presentations, the most common being the ability to invade the intestinal cells. The presence of several virulence genes, along with the host immune status, can modulate the presentation. Therefore, in this study, an attempt was made to detect virulence genes in *Shigella* species by multiplex PCR.

Antibiotic therapy is recommended in shigellosis to prevent complications and the spread of disease. Over the last few decades, *Shigella* has demonstrated unique ability to acquire horizontally-transferred genetic material, thereby making previously-efficacious drugs, like sulphonamides, tetracycline, ampicillin, and cotrimoxazole largely ineffective. The situation recently is further worsened by the emergence of multidrug-resistant strains worldwide. Therefore, the present study was also carried out to correlate the antibiotic susceptibility pattern of *Shigella* isolates with the clinical features.

## MATERIALS AND METHODS

The study was undertaken at the Department of Microbiology in JIPMER, Puducherry, India, from October 2010 to March 2012. The Ethics Committee of the institute approved the study.

A total of 33 children were included in the present study based on the inclusion criteria: children aged less than 12 years, either sex, those who came to hospital with symptoms of diarrhoea (passing stools at least 3 times in 24 hours) or dysentery (bloody stools or mucoid stools), and stool culture positive for *Shigella* species. Informed and written consents were obtained from the parents or guardians.

### Collection and processing of stool specimens

Stool samples were plated on MacConkey and xylose lysine deoxycholate agar (Himedia, Mumbai, India). Also, the sample was inoculated in Selenite F enrichment broth and subcultured after 18 hours on the abovementioned plates. Identification of *Shigella* species was done using standard biochemical tests and confirmed using antisera (Denka-Seiken, Tokyo, Japan).

### Antibiotic susceptibility pattern of the strains isolated

Antibiotic susceptibility testing was done using Kirby-Bauer disc diffusion method (3) for the antibiotics: ampicillin 10 µg, trimethoprim-sulphamethoxazole 1.25/23.75 µg, ciprofloxacin 5 µg, ceftriaxone 30 µg, tetracycline 30 µg, and chloramphenicol 30 µg (Himedia Laboratories, Mumbai). The minimum inhibitory concentration (MIC) for ciprofloxacin and ceftriaxone were performed using Epsilometer test (E-test) strips according to the manufacturer's instructions (AB Biomeriuex, India). The inoculum for the susceptibility testing and the interpretation were done as per CLSI (Clinical Laboratory Standards Institute) guidelines (3). ATCC *Escherichia coli* 25922 was used as the control for interpretation of zone diameters. Combination disc method according to CLSI guidelines was used in order to detect ESBL production in ceftriaxone-resistant *Shigella* isolates.

### Total DNA extraction

The rapid boiling method with minimal modifications was used for total DNA extraction from culture isolates (4). Briefly, the procedure was this: 5-6 colonies of *Shigellae* were picked from overnight growth and suspended in 100 µL of sterile molecular-grade water and was boiled at 100 °C for 10 minutes, following which flash-cooling was done on ice for 5 minutes. The suspension was then centrifuged at 10,000×g for 5 minutes, and the supernatant containing the DNA was stored at −20 °C till further use.

### PCR for detection of virulence genes from *Shigella* isolates

The primers used in this study for detection of virulence genes are mentioned in Table 1 (5,6). The multiplex assay was standardized with 0.3 µM concentrations of Shig and ShET2 primers and 0.5 µM concentrations of ShET1B primers. Briefly, 25 µL reaction volume consisted of 12.5 µL of master mix (Mix, Bangalore Genei/Merck), 4 µL of sterile nuclease-free water, 0.75 µL each of Shig and ShET2 primers, 1.25 µL of ShET1B primer and 3 µL of template DNA. For other primers, simplex PCR was performed.

The DNA amplification was carried out in Corbett Thermocycler, using the cycling conditions described in a previous study (5). The PCR was also done on five strains each of *Salmonella* species and *Aeromonas* species.

### PCR for ESBL detection

The primers encoding the ESBL genes: TEM, CTX, and SHV, which were used in this study, are mentioned in Table 2 (7,8). Multiplex PCR was done for the detection of CTX and SHV genes. Simplex PCR was done to detect TEM gene.

**Table 1.** Primers for detection of virulence genes (5,6)

Gene targeted	Primer	Sequence	Product-size (bp)
*set*1A	ShET1A	F:TCACGCTACCAT CAA AGA	309
		R:TATCCCCCTTTGGTGGTA	
*set*1B	ShET1B	F:GTGAACCTGCTGCCGATATC	147
		R:ATTTGTGGATAAAAATGACG	
*ial*	Ial	F:CTG GAT GGT ATG GTG AGG	320
		R:GGAGGCCAACAATTATTTCC	
*ipa*H	Shig	F:TGGAAAAACTCAGTGCCTCT	423
		R:CCAGTCCGTAAATTCATTCT	
*stx*	Stx	F:CAGTTAATGTGGTTGCGAAG	895
		R:CTGCTAATAGTTCTGCGCTC	
*sen*	ShET2	F:ATGTGCCTGCTATTATTTAT	799
		R:CATAATAATAAGCGGTCGC	

### Multiplex PCR for CTX and SHV

The multiplex PCR was done to identify *bla*SHV and *bla*CTX-M genes simultaneously. The reaction mixture and the cycling conditions described in a previous study were followed in this study (7).

### PCR for TEM

The PCR for TEM was performed using the conditions described earlier (8).

The resulting PCR products were documented on 1.5% agarose gel.

### Sequence analysis

Amplified products were sequenced to identify the virulence genes and β-lactamase resistance genes by the Macrogen Inc., Seoul, Korea. The BLASTN program (www.ncbi.nlm.nih.gov/BLAST) was used for database searching.

### Clinical profile of the study population

The clinical features of the children showing their stool positive for *Shigella* species were collected from their parents, using a structured questionnaire. The questionnaire included: age, sex of the child, number of times stool passed watery diarrhoea or associated with blood and mucus, dehydration, fever, abdominal pain, seizures, oliguria, pallor, and electrolyte abnormalities.

### Statistical analysis

GraphPad Instat software was used for statistical analysis. Fisher's exact test was used for comparing the virulence gene expression, age and gender distribution, and antibiotic resistance pattern among strains causing mild and severe disease; a p value of <0.05 was considered significant.

## RESULTS

A total of 1,424 stool specimens were collected, of which 580 were from children aged less than 12 years during the study period (October 2010 to March 2012). A total of 33 strains of *Shigella* species were isolated from 580 children, thus showing a prevalence of 6% *Shigellae* among the paediatric population attending hospital. The commonest species isolated was *S. flexneri* (79%), followed by *S. sonnei* (21%).

### Antibiotic susceptibility pattern of the strains isolated

Kirby-Bauer disc diffusion method demonstrated marked drug resistance in *Shigella* isolates to ampicillin (48%), tetracycline (88%), cotrimoxazole (84%), and chloramphenicol (12%). The method also demonstrated ciprofloxacin resistance in 70% and ceftriaxone resistance in 9% of *Shigella* isolates. MIC values by E-test method for ciprofloxacin and ceftriaxone are shown in Table 3 and 4. Resistance to more than 3 drugs was noted in 70% of *Shigella* isolates.

### Detection and distribution of virulence genes in *Shigella* isolates

**Figure 1.**
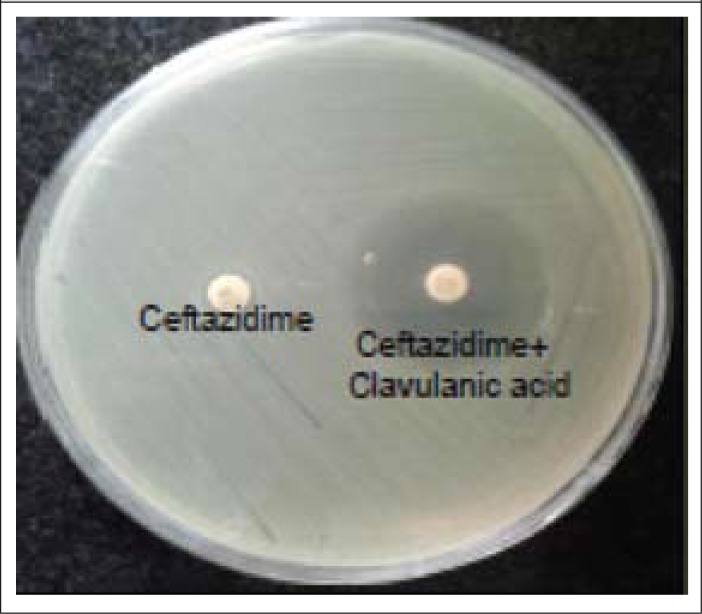
Combination disc method for ESBL detection

Multiplex PCR detected *ipa*H gene in all 33 *Shigella* isolates while it detected *ial*, *set*1A, *set*1B, and *sen* in only 28, 16, 16, and 20 isolates respectively. The method did not detect *stx* in any of the strains isolated. *Set*1A and *set*1B were found only in *S. flexneri.* DNA extracted from *Salmonella* species, and *Aeromonas* species did not show amplification for any of the above genes (Figure 1).

**Table 2.** Primers for detection of ESBL genes (7,8)

Primer	Sequence	Product-size (bp)
SHV-F	5’ATT TGT CGC TTC TTT ACT CGC 3’	1,018 bp
SHV-R	5’TTT ATG GCG TTA CCT TTG ACC 3’	
CTXMU-1	5’ATG TGC AGY ACC AGT AAR GT 3’	544 bp
CTXMU-2	5’TGG GTR AAR TAR GTS ACC AGA 3’	
TEM F	5’ATA AAA TTC TTG AAG ACG AAA 3’	1,076 bp
TEM R	5’GAC AGT TAC CAA TGC TTA ATC 3’	

**Table 3.** MIC of ceftriaxone by E-test method

Antibiotic	No. (Sensitive)	No. (Resistant)	No. (Intermediate)	MIC in µg/mL				
				≤0.016	0.023	0.032	0.38	>256
Ceftriaxone (n=33)	32	1	0	25	5	1	1	1

**Table 4.** MIC of ciprofloxacin by E-test method

Antibiotic	No. (Sensitive)	No. (Resistant)	No. (Intermediate)	MIC in µg/mL								
				≤1	1.5	2	3	4	6	8	12	>32
Ceftriaxone (n=33)	3	23	7	3	2	1	4	9	5	6	1	2

### ESBL detection

**Figure 2.**
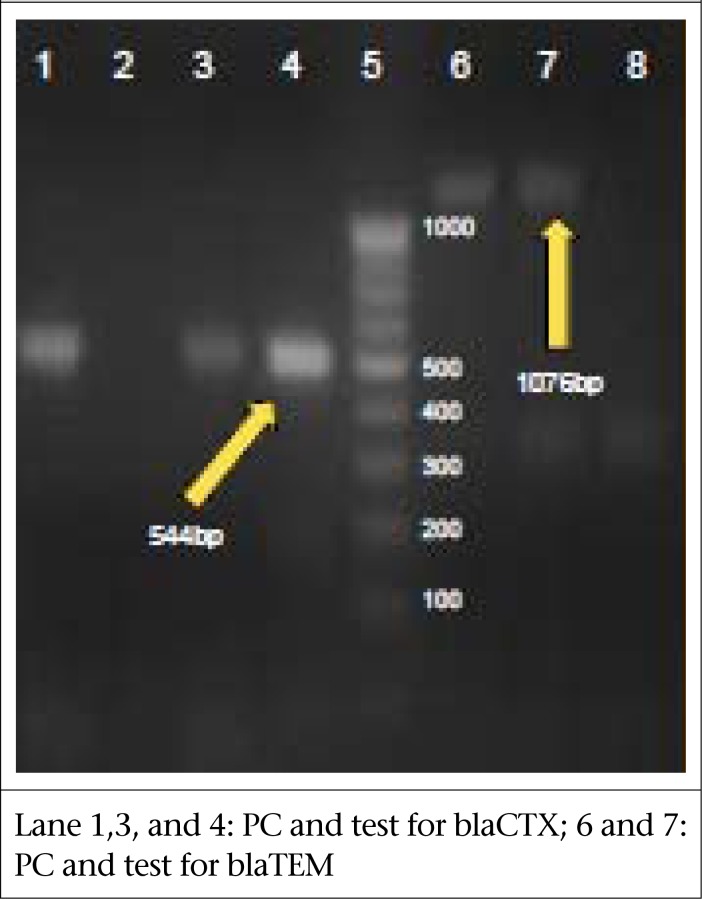
Multiplex PCR for ESBL genes

**Figure 3.**
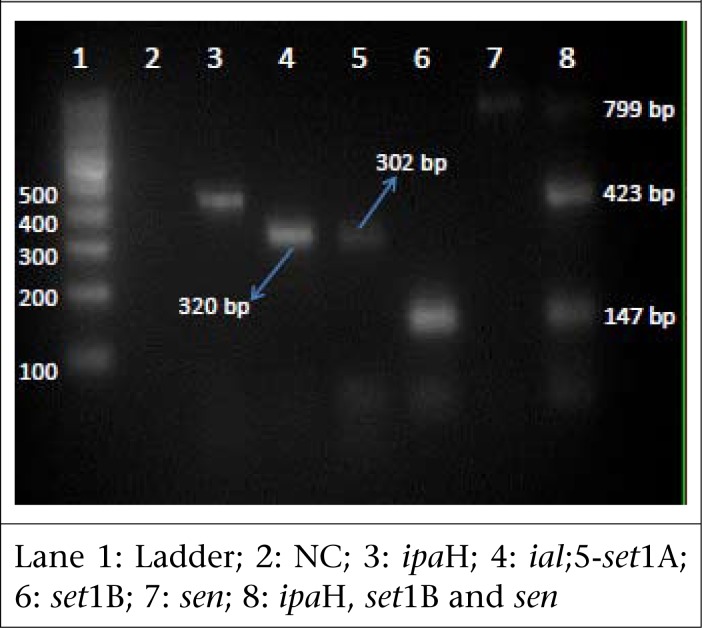
Multiplex PCR for virulence genes

Combination disc method showed all three ceftriaxone-resistant *S. flexneri* strains to be positive for ESBL production (Figure 2). Multiplex PCR for *bla*CTX and *bla*SHV genes showed 2 of these strains to be positive for *bla*CTX gene, with a band-size of 544 bp. Simplex PCR for *bla*TEM beta-lactamase showed the third strain to be positive for TEM, with a band-size of 1,018 bp (Figure 3).

### Sequencing of the amplified products

The sequencing results indicate that, out of the two strains harbouring the plasmid-encoded *bla*CTX genes, one matched the sequence of CTX-M-15 (with 99% identity; AC-FJ997866.1). The other strain harboured *bla*CTX which matched that of CTX-M-14 (with 99% identity; AC JF779678.1). The third ceftriaxone-resistant strain harboured a chromosomally-encoded *bla*TEM gene, whose sequence matched TEM-1 (with 99% identity; AC-HQ665010.1).

### Clinical profile of the study population

In the present study, shigellosis was found to be more prevalent in children (6 %) than in adult cases (1%). A total of 27 out of 33 *Shigella* strains (81%) were isolated from children aged less than 5 years. The average duration of the presenting illness was 7 days, with 2 days and 2 months being the least and maximum duration of presentation; 19 (58%) out of 33 children presented with dysentery with either blood or mucus in the stool while the rest 14 (42%) children presented with watery diarrhoea. Abdominal pain, tenesmus, fever, dehydration, decreased urine output, vomiting, and pallor were the other features present in 6 (18%), 3 (9%), 17 (51%), 4 (15%), 2 (6%), 6 (18%), and 1 (3%) respectively. Three children (9%) developed febrile seizures. None of the children had any feature of meningitis and arthritis.

## DISCUSSION

The frequency of different species of *Shigella* varies in different regions of the world. In this study, *S. flexneri* was the most common species isolated. Similar findings were noted in a study carried out earlier in our hospital (9). Previous studies done in India, Egypt, Iran, and a multicentre study by Seilden *et al*. done in China, Viet Nam, Bangladesh, Pakistan, Indonesia, and Thailand have also shown *S. flexneri* as the most common species, except in Thailand where *S. sonnei* was the most common species isolated (1,10-12).

We noted a very high resistance to ciprofloxacin in this study, which is similar to a previous study done in this department (9) and also to the other studies carried out elsewhere (2,13,14). Emergence of fluoroquinolone resistance has limited the number of antibiotics, like ceftriaxone, azithromycin, and pivmecillinam for use in shigellosis. Unfortunately, resistance to the third-generation cephalosporins in *Shigella* species has been reported from several studies, and the same has been observed in this study (15-17).

Resistance to 3 or more antimicrobials were shown in previous studies done in North India and Viet Nam (10,18). In this study, multiple drug resistance was observed in more than two-thirds of *Shigella* isolates, regardless of the serotype.

All the 33 *Shigella* strains tested by multiplex PCR was positive for *ipa*H gene. Similar findings have been shown in a study carried out in Malaysian *Shigella* species by Thong *et al*. (5). Multiple copies of this gene being present on both plasmids and chromosomes may explain the gene being tested positive in all strains (19).

Most isolates also showed the presence of *ial* gene. A study by Thong *et al*. showed 40% of strains possessing *ial* gene. The *ial* gene being located only on *inv* plasmid, unlike *ipa*H which is present both on plasmid and chromosome, is prone to spontaneous deletions (5,19). This may probably explain less number of strains harbouring this gene when compared with *ipa*H gene.

The *Shigella* enterotoxin (ShET1) genes *set*1A and *set*1B encoded chromosomally were found in almost half of the *S. flexneri* strains. Both of these genes were present in the same isolates; the finding supported the previous observations and showed that these genes exist as tandem in the *Shigella* genome (5). A study by Farfan *et al*. has shown that nearly 30% of the *Shigella* strains harboured *set* gene (20). Previous studies have shown that these genes are most commonly present in *S. flexneri* 2a. Although no further typing of *S. flexneri* was performed in our study, ShET 1 was found only in *S. flexneri* strains and not in *S. sonnei*, thus confirming the previous findings.

In this study, three ceftriaxone-resistant *S. flexneri* strains isolated showed the presence of CTX-M-15, CTX-M-14, and TEM-1. There are several other reports of ESBL-producing *Shigella* from India (15-17). In the recent years, various ESBL-producing *Shigellae* were also reported from Korea (CTX-M-14), Argentina (CTX-M-2), Viet Nam (CTX-M-15, and CTX-M-24), France, and Turkey (21-24).

World over, the major burden of shigellosis is contributed by children aged less than five years. In this study also, the majority of *Shigella* species were isolated from children aged less than 5 years. The underdeveloped immune system and the fact that the faeco-oral transmission is more common in this age-group make this age-group more susceptible. These findings were in contrast with a study by Ghosh *et al*. from Kolkata, India, where shigellosis was more common in children aged above 5 years (25).

Three children (2 with *S. flexneri* and 1 with *S. sonnei*) aged less than five years developed seizures during their hospital stay. Hyperthermia was noted in all three children. Hyponatraemia was seen in one child, and severe dehydration was seen in another. Other risk factors, like hypoglycaemia, hyperkalaemia, and elevated serum creatinine concentrations, which were shown to be associated with seizures in previous studies, were not detected in our study (26-28). This supports the findings of a study done by Khan *et al*. that less than five years old children with fever and metabolic abnormalities were at increased risk of seizures in shigellosis (26). This finding also suggests the fact that central nervous system manifestations are not always related to Shiga toxin, which is produced in appreciable amounts only by *S. dysenteriae*. Therefore, early interventions to reduce fever and correction of metabolic abnormalities in children can help in preventing such complications. Persistent diarrhoea was seen in a child with Grade III protein-energy malnutrition in this study. No deaths were reported in this study.

### Conclusions

Dysentery was the most common presentation, followed by fever and abdominal pain. Children aged less than five years having fever and metabolic abnormalities, like hyponatraemia, are at an increased risk of seizures in shigellosis. Resistance to ciprofloxacin and ceftriaxone is an alarming situation warranting proper usage of antimicrobial agents and continuous monitoring of antimicrobial resistance.
